# Vaginal chlamydial clearance following primary or secondary infection in mice occurs independently of TNF-α

**DOI:** 10.3389/fcimb.2013.00011

**Published:** 2013-03-11

**Authors:** Sangamithra Kamalakaran, Bharat K. R. Chaganty, Rishein Gupta, M. Neal Guentzel, James P. Chambers, Ashlesh K. Murthy, Bernard P. Arulanandam

**Affiliations:** ^1^Department of Biology, South Texas Center for Emerging Infectious Diseases, University of Texas at San AntonioSan Antonio, TX, USA; ^2^Center of Excellence in Infection Genomics, The University of Texas at San AntonioSan Antonio, TX, USA; ^3^Department of Pathology, Midwestern UniversityDowners Grove, IL, USA

**Keywords:** TNF-α, *Chlamydia*, granzyme, primary and secondary genital infection, upper genital tract pathology

## Abstract

The role of TNF-α in chlamydial clearance is uncertain. Antibody-mediated depletion of TNF-α in mice and guinea pigs has been shown not to significantly affect chlamydial clearance, whereas production of TNF-α in addition to IFN-γ from T cells has been shown to correlate with enhanced clearance. The aim of our study is to evaluate the mechanistic role of TNF-α in clearance of primary and secondary chlamydial infection from the genital tract (GT) using C57BL/6 TNF-α deficient (TNF-α^−/−^) and wild type (WT) mice. Chlamydial shedding from the lower GT was evaluated following primary and secondary intravaginal challenge. Also, antibody and antigen specific cytokine responses were analyzed from the infected GT and spleens, and oviduct pathology determined to analyze the role of TNF-α in upper GT pathological sequelae. MHC II^−/−^ mice, known to display muted adaptive immune responses and failure to resolve genital chlamydial infections, were used as a negative control. Following both primary and secondary genital chlamydial infection, TNF-α^−/−^ mice exhibited elevated granzyme B production, but similar IFN-γ and antibody responses. Importantly, absence of TNF-α did not significantly alter the resolution of infection. However, TNF-α^−/−^ mice displayed significantly reduced upper genital tract (UGT) pathology compared to WT mice. This study demonstrates mechanistically that optimal chlamydial clearance following primary and secondary chlamydial genital infection can occur in the complete absence of TNF-α, and considered with the reduction of upper GT pathology in TNF-α^−/−^ mice, suggests that targeted induction of anti-chlamydial TNF-α responses by vaccination may be unnecessary, and moreover could be potentially pathogenic.

## Introduction

*Chlamydia trachomatis*, a Gram-negative obligate intracellular bacterium, is the leading cause of bacterial sexually transmitted diseases (STDs) worldwide. In females, the majority of cases are initially asymptomatic and, when left untreated, may ascend to the upper genital tract (UGT) and cause severe sequelae including pelvic inflammatory disease, ectopic pregnancy, and infertility (Morrison and Caldwell, 2002; Debattista et al., 2003; Brunham and Rey-Ladino, 2005; Meldrum et al., 2007). Although there is efficacious antimicrobial therapy (Beale et al., 1991; Beale and Upshon, 1994; Su et al., 1999), a continual increase in the incidence of chlamydial STD argues for the development of a safe preventive vaccine (Morrison and Caldwell, 2002; Debattista et al., 2003; Brunham and Rey-Ladino, 2005).

Infection with *Chlamydia* in the genital tract (GT) induces the production of several pro-inflammatory cytokines such as TNF-α, IL-1, and IL-6, chemokines such as IL-8, and inflammatory cytokines including IL-12, IFN-γ, and IL-17 (Su et al., 1999; Brunham and Rey-Ladino, 2005; Murthy et al., 2011). Among these, IL-12 and IFN-γ have been shown previously to contribute to protective immunity against chlamydial GT infection (Brunham and Rey-Ladino, 2005). The role of TNF-α in chlamydial infections has been studied previously. Transcripts of TNF-α are found in the infected GT tissues as early as day 3 after infection (Morrison and Morrison, 2000). We and others also have shown that there is an initial surge of TNF-α production in the GTs of infected mice during the first week of infection, and that the levels gradually reduce during the subsequent weeks of infection (Darville et al., 1995, 1997; Murthy et al., 2011). TNF-α has pleiotropic effects: it is a potent chemoattractant of neutrophils and acts as a key transcription factor in the NF-kappa B and MAP kinase pathways triggering inflammation, immune cell proliferation and apoptosis (Strieter et al., 1989; Wajant et al., 2003). Specifically, it is known that apoptosis and fibrosis are due to the effects of TNF-α via caspase-8 activation (Alikhani et al., 2004), and that soluble TNF mediates fibrosis in the lung (Oikonomou et al., 2006). Importantly, we have shown recently that TNF-α production contributes to upper GT pathology following vaginal chlamydial infection in mice (Murthy et al., 2011). In that study, we also showed that TNF-α was not required for clearance of primary genital chlamydial infections. However, a detailed evaluation of compensatory immunological parameters or the role of TNF-α production in clearance of secondary genital chlamydial infections was not conducted in that study.

One of the well-recognized roles of TNF-α is in killing of intracellular microbes. There is evidence showing that the addition of recombinant TNF-α *in vitro* inhibits intracellular replication of *C. trachomatis* in human laryngeal carcinoma cells (Hep-2 cells) (Manor and Sarov, 1988). However, the effects of TNF-α in the clearance of *in vivo* chlamydial infections are uncertain. Darville et al. (2000), demonstrated that TNF-α depleted mice and guinea pigs, when infected with *Chlamydia*, show comparable clearance and levels of infection to their respective WT animal controls. However, the investigators suggested that the anti-TNF-α antibody used to deplete the cytokine might not have been sufficient for complete depletion. In contrast, Caldwell et al. suggested that TNF-α may be important for chlamydial resistance *in vivo* since TNF-α p55 receptor gene deficient mice displayed significantly enhanced chlamydial shedding, but comparable time to clearance of *Chlamydia muridarum* (*C. muridarum*), when compared to corresponding WT mice (Perry et al., 1999b). More recently, reduced chlamydial shedding from the GT on day 10 after challenge in vaccinated mice was found to correlate better with frequencies of T cells producing both TNF-α and IFN-γ, than those producing IFN-γ alone, thus suggesting that TNF-α may be one of the effector cytokines contributing to optimal protection in *Chlamydia* vaccinated mice (Yu et al., 2011). Collectively, these disparate observations emphasize the need for a detailed evaluation of the role of TNF-α in clearance of genital chlamydial infections.

In this study, we evaluated the host responses to *C. muridarum* in the absence of TNF-α following both primary and secondary intravaginal infection, using TNF-α^−/−^ and the corresponding WT C57BL/6 mice. We hypothesized that TNF-α would contribute to chlamydial clearance and thus TNF-α^−/−^ mice would display significantly reduced chlamydial clearance compared to WT mice. A known mouse model of MHC II gene deficiency (MHC II^−/−^ mice), which has been determined previously to be significantly deficient in chlamydial clearance (Murthy et al., 2006), was used as a negative control for all experiments. We found that the mice deficient in TNF-α did not show significant differences in resolution of either primary or secondary *C. muridarum* infection. Additionally, the cell mediated and humoral responses were generally comparable in TNF-α^−/−^ and WT C57BL/6 mice. In contrast, oviduct pathology in TNF-α^−/−^ mice was significantly less than in WT animals, suggesting that TNF-α may play a role in triggering inflammatory responses leading to the oviduct pathology.

## Materials and methods

### *Chlamydia muridarum* and mice

*Chlamydia muridarum* Nigg (*C. muridarum*) strain was grown in HeLa 229 cells, and elementary bodies (EBs) were obtained using a Renografin gradient separation method as described previously (Wang et al., 2009; Nagarajan et al., 2011). UV inactivated organisms were generated by subjecting purified EBs to 45 min of UV irradiation using an UV crosslinker. Female, 4–6 week old C57BL/6 WT mice and breeding pairs of TNF-α deficient (TNF-α^−/−^) and MHC class II deficient (MHC II^−/−^) mice were purchased from the Jackson Laboratory and maintained at the University of Texas at San Antonio and Midwestern University. Food and water were supplied *ad libitum* and all experimental procedures followed the guidelines of the Institutional Animal Care and Use Committee (IACUC) at both institutions.

### Intravaginal infection of mice and monitoring of bacterial shedding

WT, TNF-α^−/−^ and MHC class II^−/−^ mice received 2.5 mg per mouse of Depoprovera (medroxy-progesterone acetate) subcutaneously at 10 and 3 days before vaginal challenge in order to render the mice anestrous and more receptive to the genital infection. The mice were then challenged with 5 × 10^4^ inclusion-forming units (IFU) of *C. muridarum* contained in 10 μ l of sucrose-phosphate-glutamate buffer placed into the cervico-vaginal vault. The course of infection was followed by swabbing the cervico-vaginal vault every 3 days following inoculation. For secondary challenge, animals that recovered from primary infection were administered 0.03 mg Doxycycline intraperitoneally (i.p) in 100 μl phosphate-buffered saline (PBS) (Jupelli et al., 2011) each day on days 60–63 following primary challenge to ensure clearance of the primary infection. Mice were rested for 1 week and rechallenged with 5 × 10^4^ EBs following Depoprovera treatment, as described above. To assess bacterial burdens, swabs were collected in Eppendorf tubes containing 4 mm glass beads (Kimble, Vineland, NJ) and 500 μl of sterile SPG buffer. The tubes were vortexed for 1 min, and swab material was plated onto HeLa cells grown on coverslips in 24-well plates and incubated for 30 h. The infected HeLa cells were fixed with 2% paraformaldehyde and permeabilized with 2% saponin. Cells were washed using PBS and incubated for 1 h with Dulbecco's modified Eagle's medium containing 10% fetal bovine serum to block non-specific binding. Thereafter, cells were washed and incubated with polyclonal rabbit anti-*Chlamydia* antibody for 1 h and then incubated for an additional 2 h with goat anti-rabbit immunoglobulin conjugated to fluorescein isothiocyanate (Sigma, St. Louis, MO) along with Hoechst nuclear stain. The treated coverslip cultures were then washed and mounted on Superfrost microscope slides (Fisher) using Fluorsave reagent (Calbiochem, La Jolla, CA), and the chlamydial IFUs were visualized using a Zeiss Axioskop 2 Plus research microscope (Zeiss, Thornwood, NY).

### Evaluation of cytokine and antibody responses

Eight days after challenge with *C. muridarum*, mice (3 per group) were euthanized and GTs collected and homogenized individually in PBS containing protease inhibitor. Supernatants were collected after centrifugation at 5000 rpm for 10 min and analyzed. In some experiments, 14 days after primary infection with *C. muridarum*, mice (3 per group) were euthanized, spleens collected, and single cell suspensions made. Splenocytes (10^6^ cells/well) were stimulated with 10^4^ UV irradiated EB, or the unrelated protein hen egg lysozyme (HEL), or medium alone, and incubated for 72 h. In experiments where splenocytes were collected at 12 days after secondary challenge, it was assumed that the cells would have been restimulated *in vivo*, and therefore additional *in vitro* antigenic stimulation was not provided. Supernatants were collected and assayed for IFN-γ using BD OptEIA™ kits (BD Pharmingen, San Diego, CA); and for TNF-α and granzyme B using Ebioscience kits (San Diego, CA). The levels of respective cytokines were quantified by measuring the absorbance at 630 nm using a μ Quant ELISA plate reader (BioTek Instruments, Winooski, VT). Antibody ELISA tests were conducted on sera collected at 40 days after primary infection with live EB and at 12 days after reinfection with live EB. Microtiter plates were coated with 10^5^ UV-EB, or the unrelated antigen HEL, and incubated overnight at 4°C. Serial dilutions of sera were performed in the plates followed by incubation for 2 h. After 3 washes, goat anti-mouse total Ig (H+L), IgG2a, IgG2b, or IgG2c conjugated to horseradish peroxidase (HRP) was added as secondary antibody (Southern Biotechnology). Tetramethylbenzidine was used as substrate and the resulting absorbance quantified at 630 nm using a μ Quant ELISA plate reader (BioTek Instruments, Winooski, VT).

### Estimation of uterine horn and oviduct pathology

UGT pathology was evaluated on day 70 post-challenge. Mice (6 per group) were euthanized, GTs removed and examined for the presence of gross hydrosalpinx. The tissues were then aligned next to a standard metric ruler and photographed from a fixed distance using a 6 mega-pixel Fuji F10 camera. Images were stored at high resolution and printed on sheets (A4 size) of paper. Dilated oviducts measuring >0.5 mm in diameter were used as an indicator of hydrosalpinx. When multiple oviduct loops were present, the one with the greatest diameter was reported. For uterine horns, the average of the greatest cross-sectional diameter of each 5-mm longitudinal section was reported. The baseline normal mouse oviduct diameter was determined to be 0.5 mm and normal uterine horn diameter to be 1 mm by prior analysis of a group of age-matched naïve mice. Pooled data from two experiments was analyzed for statistically significant differences.

### Statistical analyses

Sigma Stat (Systat Software Inc., San Jose, CA) was used to perform all tests of significance. Analysis of variance (ANOVA; Systat, CA) was used for all comparisons. Differences between groups were considered statistically significant if *P*-values were ≤0.05. All experiments were repeated at least twice, and each experiment was analyzed independently. In experiments where oviduct and uterine horn diameter data are shown as a composite of two experiments, the indicated significant difference holds true when the experiments are analyzed individually.

## Results

### Effect of TNF-α on clearance of *chlamydia muridarum* primary genital infection

**Figure 1:**
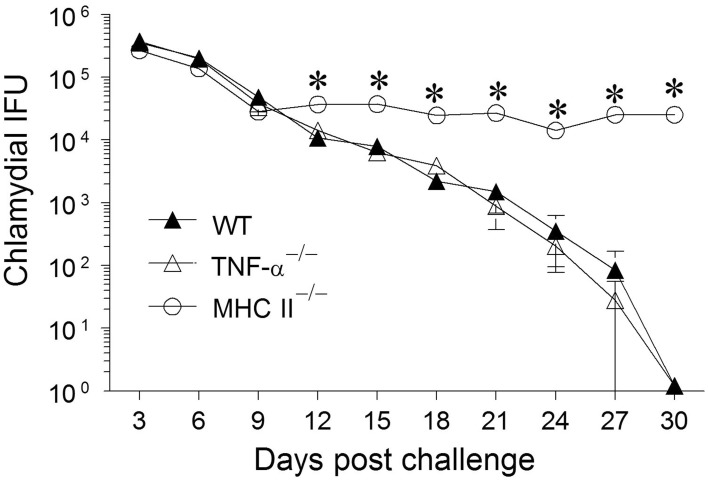
**Chlamydial clearance following primary genital infection with *C. muridarum*.** Groups of C57BL/6 WT, TNF-α^−/−^, and MHC II^−/−^ mice (*n* = 6) were swabbed and analyzed every 3 days over a 30 day period for numbers of chlamydial IFU recovered following primary genital infection with *C. muridarum*. Results are represented as mean ± SEM of chlamydial recovery per mouse group at each time point. ^*^Significant (*p* ≤ 0.05, Two-Way ANOVA) differences between MHC II^−/−^ and WT or between MHC II^−/−^ and TNF-α^−/−^ mice at the indicated time-periods. Results are representative of two independent experiments.

Groups (*n* = 6) of WT, TNF-α^−/−^, and MHC II^−/−^ mice were challenged with 5 × 10^4^ IFUs of *C. muridarum* and chlamydial numbers in vaginal swabs were monitored every 3 days for 30 days. WT mice shed high numbers of bacteria at early time-periods and displayed a progressive reduction in vaginal chlamydial shedding with complete clearance by day 30 after primary infection (Figure 1). The kinetics of resolution of infection in TNF-α^−/−^ mice was comparable to that in WT mice. As expected, MHC II^−/−^ mice continued to display a high level of chlamydial shedding throughout the period of monitoring and none of the mice in this group had cleared the infection at day 30 after primary infection. At day 12, and every time-point thereafter, WT and TNF-α^−/−^ mice displayed a significant reduction in vaginal chlamydial shedding when compared to MHC II^−/−^ mice. These results suggest that the presence of TNF-α is not required, nor does it significantly affect the clearance of primary genital infection with *C. muridarum*.

### Effect of TNF-α on immune responses to *chlamydia muridarum* primary genital infection

**Figure 2:**
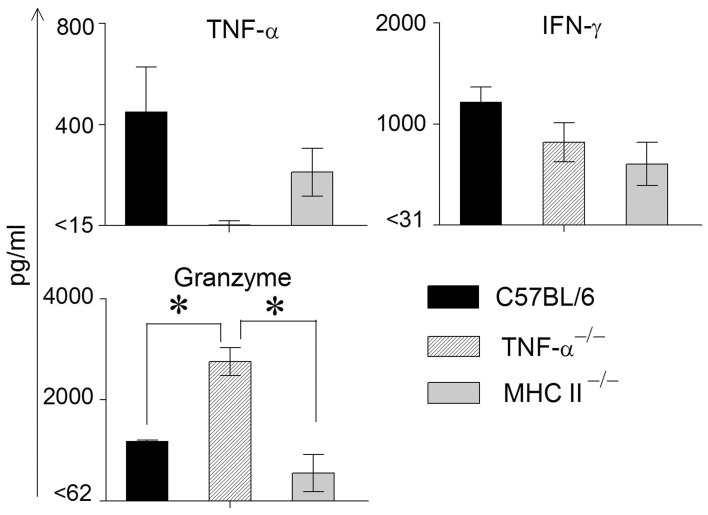
**Cytokine production in genital tracts following primary genital infection with *C. muridarum*.** Genital tracts of individual mice from each group (*n* = 3) on day 8 after primary genital challenge with *C. muridarum* were harvested, homogenized, and TNF-α, IFN-γ, and granzyme B measured. Results are represented as mean ± SEM for each cytokine per mouse group. ^*^Significant (*p* ≤ 0.05, ANOVA) difference between the indicated groups. Results are representative of two independent experiments.

We have found previously that the granzyme and perforin pathway contributes to the GT pathology following chlamydial infection (Murthy et al., 2011). To evaluate any compensatory effects of granzyme in mice lacking TNF-α production, we also evaluated the production of granzyme in these assays. We have observed previously that the levels of TNF-α and granzyme B peaked at approximately 1 week following primary genital chlamydial challenge in the WT mouse GT, but returned to normal levels as weeks progressed (Murthy et al., 2011). Therefore, GTs from infected WT, TNF-α^−/−^ and MHC II^−/−^ mice were removed on day 8 post challenge and analyzed for IFN-γ, TNF-α and granzyme B production (Figure 2). TNF-α production by WT mice was greater, albeit not significantly, than that exhibited by MHC II^−/−^and as expected not detectable in TNF-α^−/−^ mice. WT mice displayed the highest levels of IFN-γ production, followed by TNF-α^−/−^ and MHC II^−/−^ mice, respectively, although no significant differences were observed. On the other hand, granzyme B levels were comparable between WT and MHC II^−/−^ mice, and both were significantly lower compared to that in TNF-α^−/−^ animals.

**Figure 3:**
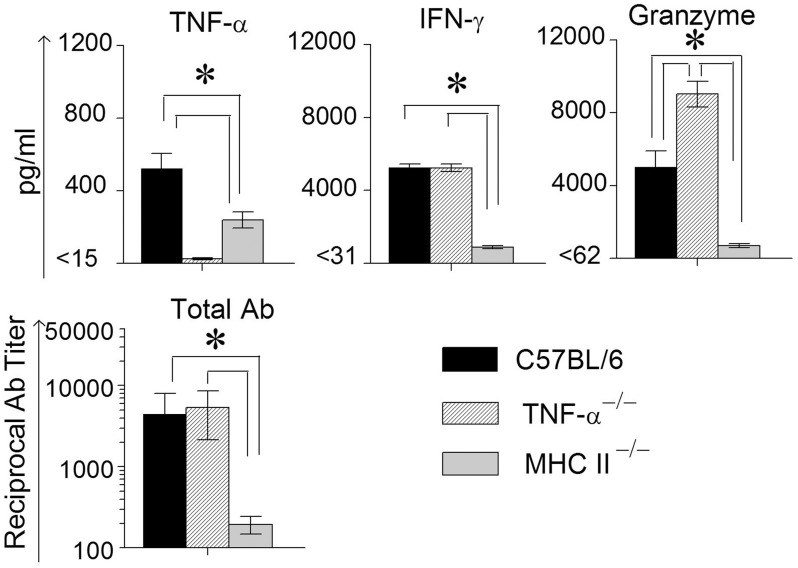
**Antigen specific cytokine responses measured from splenocytes and serum antibody response after primary genital infection with *C. muridarum*.** Spleens from different groups of mice were collected at 14 days after primary genital challenge with *C. muridarum* and levels of IFN-γ, TNF-α and granzyme B produced by splenocytes were measured from supernatants 72 h after *in vitro* stimulation of the splenocytes with UV-inactivated EBs, unrelated antigen, or medium alone. Results are represented as mean ± SEM for each cytokine per mouse group. ^*^Significant (*p* ≤ 0.05, ANOVA) difference between the indicated groups. Results are representative of two independent experiments. Total Ig from the different groups of mice were measured against UV inactivated EBs in serum collected from infected animals on day 40 after primary genital challenge with *C. muridarum*. Results are represented as mean ± SEM of reciprocal titers corresponding to 50% maximum binding. ^*^Significant (*p* ≤ 0.05, ANOVA) difference between the indicated groups. Results are representative of two independent experiments.

In order to measure Ag-specific cellular cytokine production, spleens were collected from WT, TNF-α^−/−^, and MHC II^−/−^ mice on day 14 following primary genital challenge with *C. muridarum*, single cells prepared and stimulated with UV inactivated EBs and supernatants analyzed for the same three cytokines. As shown in Figure 3, WT mice displayed significantly higher levels of TNF-α production when compared to MHC II^−/−^ mice, and both these groups produced significantly greater TNF-α than TNF-α^−/−^ mice, which as expected did not display TNF-α production. High, and comparable, levels of Ag-specific IFN-γ production were observed in WT and TNF-α^−/−^ mice, and both displayed significantly higher levels than MHC II^−/−^ animals. Granzyme B production in WT mice was significantly higher than in MHC II^−/−^ mice, and the levels in TNF-α^−/−^ mice were significantly greater than that in either WT or MHC II^−/−^ mice. In all groups of mice, splenocytes stimulated with the unrelated antigen HEL and media alone produced low minimal levels of TNF-α, IFN-γ, and granzyme B (data not shown).

Mice bled at day 40 after primary challenge were measured for antibody levels (Figure 3). Robust and comparable serum antibody responses for total IgG (Figure 3) and IgG1, IgG2b, and IgG2c isotypes (data not shown) were observed in WT and TNF-α^−/−^ mice, and both displayed significantly higher titers of all the measured antibody isotypes when compared to MHC II^−/−^ mice which, as expected, displayed minimal antibody responses. Serum antibody responses against the unrelated antigen HEL was undetectable (data not shown). Together, these results clearly indicate that the absence of TNF-α during the chlamydial infection process does not significantly affect IFN-γ production in the infected GT, or *Chlamydia*-specific IFN-γ and antibody production. However, granzyme B production in the infected GT and from Ag-specific splenocytes increased significantly in mice deficient in TNF-α production.

### Evaluation of the effect of TNF-α on upper genital tract pathological sequelae following *chlamydia muridarum* primary genital infection

**Figure 4:**
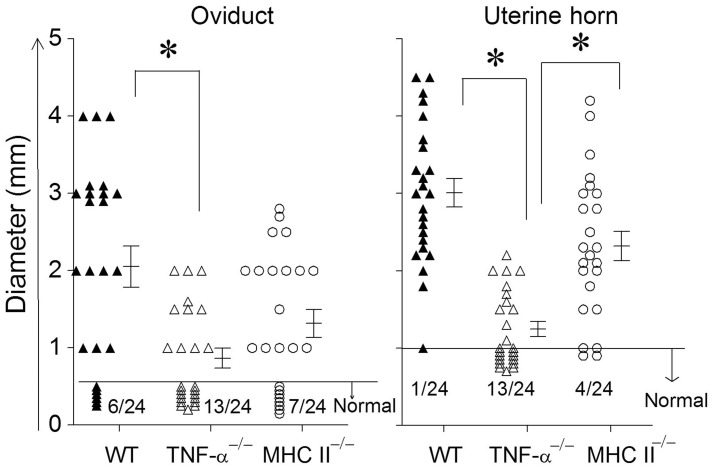
**Upper genital tract pathological sequelae following primary genital infection with *C. muridarum*.** Genital tract tissues from animals were harvested at 80 days after primary genital challenge with *C. muridarum* and pathology was evaluated by measuring the cross sectional diameter of both the oviduct and uterine horn as an inductor of the severity of inflammation. Results are represented as individual oviduct or uterine horn cross-sectional diameter and the mean ± SEM for each mouse group also is shown. A horizontal line at 0.5 mm for oviduct diameter and 1 mm for uterine horn diameter represents the distinction between normal and dilated. The ratio of the number of oviducts or uterine horns that were normal to the total number examined are indicated above the X-axis for each mouse group. ^*^Significant (*p* ≤ 0.05, ANOVA) difference between the indicated groups, as analyzed from pooled data. Results are representative of two independent experiments.

The development of oviduct and uterine horn dilatation are characteristic sequelae to genital chlamydial infection in WT mice. At day 80 after bacterial challenge, mice were euthanized and GTs were collected for evaluating pathology. GTs were photographed and pathology was scored by measuring the cross sectional diameter of the tissue (Murthy et al., 2011). The incidence of oviduct dilatation in WT (18/24) and MHC II^−/−^ (17/24) mice was significantly higher than in TNF-α^−/−^ (11/24) mice (Figure 4). Additionally, the degree of dilatation in the affected oviducts was significantly greater in WT, when compared to TNF-α^−/−^ mice, with an intermediate level of dilatation in MHC II^−/−^ mice which was not significantly different from the other groups. The incidence of uterine horn dilatation in WT (23/24) and MHC II^−/−^ (20/24) mice was significantly higher than in TNF-α^−/−^ (11/24) mice. Additionally, the degree of dilatation in the affected uterine horns was significantly reduced in TNF-α^−/−^ mice when compared to either WT or MHC II^−/−^ mice. The level of uterine horn dilatation in MHC II^−/−^ mice was reduced, albeit not significantly, when compared to WT mice. Collectively, these results suggest that TNF-α produced from *Chlamydia* infected cells contributes significantly to development of UGT pathologies.

### Effect of TNF-α on secondary genital infection with *chlamydia muridarum*

**Figure 5:**
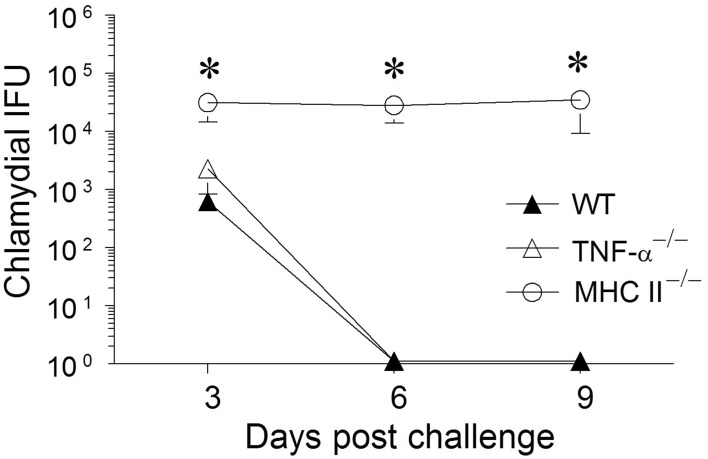
**Chlamydial clearance following secondary genital infection with *C. muridarum*.** Sixty days following primary genital infection with *C. muridarum*, mice from each animal group (*n* = 6) were treated for 4 days with Doxycycline (30 μg/mouse/day) to ensure that the animals cleared *Chlamydia*. After the Doxycycline treatment, mice were swabbed every third day for 9 days to document chlamydial clearance. All mice were then given Depo-progesterone (day 70), and 5 days later re-challenged intravaginally with 5 × 10^4^ EBs. Mice were swabbed and analyzed for chlamydial IFU recovered every third day for 9 days. Results are represented as mean ± SEM of chlamydial recovery per mouse group at each time point. ^*^Significant (*p* ≤ 0.05, One-Way ANOVA) differences between MHC II^−/−^ and WT or between MHC II^−/−^ and TNF-α^−/−^ mice at the indicated time periods. Results are representative of two independent experiments.

**Figure 6:**
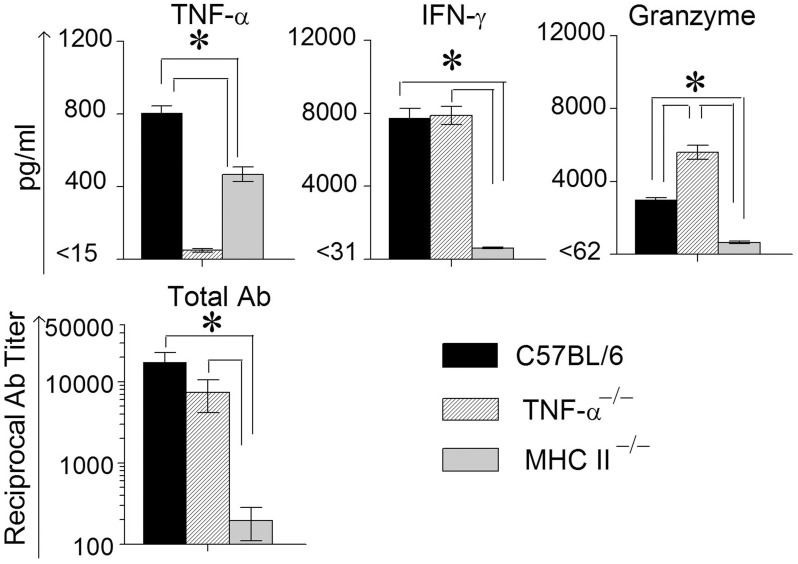
**Antigen specific cytokine responses measured from splenocytes and serum antibody response after secondary genital infection with *C. muridarum*.** Spleens from different groups of mice were collected at 12 days after secondary genital challenge with *C. muridarum*, single cells prepared, and levels of IFN-γ, TNF-α and granzyme B produced by the splenocytes were measured from supernatants 72 h after incubation in media alone. These cells were considered to be re-stimulated with antigen *in vivo*. ^*^Significant (*p* ≤ 0.05, ANOVA) difference between the indicated groups. Results are representative of two independent experiments. Total Ig from the different groups of mice were measured against UV inactivated EBs in serum collected from infected animals on day 12 following secondary genital challenge with *C. muridarum*. Results are represented as mean ± SEM of reciprocal titers corresponding to 50% maximum binding. ^*^Significant (*p* ≤ 0.05, ANOVA) difference between the indicated groups. Results are representative of two independent experiments.

Animals were treated with doxycycline for 4 days starting at 60 days after primary challenge and were swabbed to ensure that the infection was cleared in all groups of mice, especially the MHC II^−/−^ mice (data not shown). The mice were rested for a week before they were reinfected intravaginally with 5 × 10^4^ IFU of *C. muridarum*. As shown in Figure 5, all of the reinfected mice displayed high levels of chlamydial shedding at day 3 after secondary infection. WT and TNF-α^−/−^ mice rapidly cleared *C. muridarum* by day 6, whereas MHC II^−/−^ mice continued to shed high numbers (>10^4^ IFU) of the organism even on day 9 following reinfection, displaying similar kinetics as in the primary infection. At day 12 after secondary challenge, splenocytes were cultured *in vitro* for 72 h and the supernatants were analyzed for TNF-α, IFN-γ, and granzyme B. As shown in Figure 6, the antigen specific TNF-α and IFN-γ responses followed a similar trend as in primary infection, but, as expected, much higher levels of cytokines were produced. Granzyme B production between the groups also followed a similar trend, but at notably reduced levels, to that seen in primary infection. Additionally, robust serum antibody responses, including total IgG (Figure 6), IgG1, IgG2b, and IgG2c, (data not shown) against *C. muridarum* were observed in reinfected WT and TNF-α^−/−^ mice with reduced levels in MHC II^−/−^ mice. As seen with the Ag-specific cytokine responses, antibody responses following secondary infection between the different groups of mice followed a similar trend, albeit at much higher levels, to that observed following primary infection, with no significant reduction in the absence of TNF-α.

## Discussion

The role of TNF-α in the mouse model of genital chlamydial infection has not been fully resolved. Previous studies using anti-TNF-α antibody or TNF-α R1 gene deficient mice displayed a minimal role for TNF-α in clearance of primary genital *C. muridarum* infection (Perry et al., 1999b). However, there has been renewed interest in the contribution of this cytokine to protective immunity based on a recent report of correlation between T cells producing TNF-α in addition to IFN-γ and chlamydial clearance (Yu et al., 2011). In our study, we used gene knockout mice incapable of TNF-α production, and found that clearance of both primary and secondary infection was not significantly affected in the complete absence of TNF-α production. However, TNF-α deficient mice did display minimal UGT pathology, further confirming our recent demonstration of this phenomenon and suggesting that TNF-α contributes to UGT pathology (Murthy et al., 2011).

We and others have shown previously that TNF-α production within the GT is seen in the first few weeks following *Chlamydia* challenge (Darville et al., 2000; Murthy et al., 2011). Given the known pleiotropic effects of TNF-α in inducing inflammation, bacterial clearance, apoptosis, and pro-survival signals (Strieter et al., 1989; Wajant et al., 2003; Alikhani et al., 2004; Oikonomou et al., 2006), it would be reasonable to expect that this cytokine would contribute during genital chlamydial infection. TNF-α has been shown to exert these effects mainly via two receptors: TNF receptor 1 (TNFR1) and TNFR2 (Wajant et al., 2003). The results from the current study suggest that gene deficiency leading to lack of TNF-α production does not significantly affect the clearance of either primary or secondary genital chlamydial infection. This is in agreement with results from the study using anti-TNF-α antibody to deplete the cytokine (Darville et al., 2000). However, a contribution of signaling via TNFR1 to controlling chlamydial burden, without affecting time to resolution, during primary infection in the mouse GT has been documented (Perry et al., 1999b). First, the insignificant difference in chlamydial burdens between WT and TNF-α deficient mice was not due to our inability to detect such a difference since we could clearly show the elevated chlamydial burden in MHC II deficient mice. While we are unclear about the precise reason for this subtle discrepancy, we can suggest some possibilities. First, TNF-α signals via multiple receptors, and the effect of individual receptor signaling pathways may complement or oppose each other; therefore, an isolated deficiency of TNFR1 may not be the same as complete absence of TNF-α. A study comparing the individual effects of the TNF receptors may address this issue. Second, the Perry et al. study showed that TNF-α did not have any measurable effect on *in vitro* growth of *C. muridarum*, suggesting that any effect of this cytokine on chlamydial growth may be mediated indirectly by activating other cell types and cytokine production (Perry et al., 1999b). Since gene deficient mice have been known to display bystander effects on non-targeted molecules, it may be that components of the immune system other than TNF-α or TNFR1 were affected differently in these models and thereby had an impact on chlamydial burdens. For example, the levels of granzyme were significantly higher during the chlamydial infection in TNF-α deficient mice when compared to WT animals. Previous studies from our group (Murthy et al., 2011) and others (Perry et al., 1999a) have shown that perforin does not contribute significantly to chlamydial clearance. However, the levels of IFN-γ and antibody production, which have been shown to contribute to chlamydial clearance (Brunham and Rey-Ladino, 2005), were comparable between TNF-α deficient and WT animals. A similar assessment of the profiles of different components of the immune system was not conducted in the Perry et al. study (Perry et al., 1999b), making it difficult to obtain further insights into such differences.

TNF-α deficient mice displayed significantly reduced oviduct pathology when compared to WT animals following primary genital *C. muridarum* infection. This confirms our recent demonstration of the role of TNF-α in chlamydial pathogenesis in the mouse model, and is supported by evidence of a role of TNF-α in chlamydial pathologies in *Chlamydia*-induced infertility in humans (Ohman et al., 2009). Furthermore, we (Murthy et al., 2011) and others (Perry et al., 1999a) have shown previously that perforin deficient mice display significantly reduced pathology compared to wild type (WT) animals, suggesting a role for granzymeB/perforin pathway in chlamydial pathogenesis. In this study, TNF-α deficient mice displayed significantly reduced UGT pathology, but high levels of granzyme B production in infected GTs and from antigen-specific splenocytes. This suggests that the increased granzyme B production may represent a compensatory mechanism for the deficiency of TNF-α. Conversely, TNF-α may directly or indirectly negatively regulate the production of granzyme B in WT animals. Further studies will be needed to obtain detailed insights into the contribution of granzyme B in this model. The identification of a pathogenic role for TNF-α has several implications. First, TNF-α may serve as a molecular target to reduce the clinically relevant reproductive pathologies that follow genital chlamydial infections. Anti-TNF-α therapies are used in clinics currently for inflammatory bowel disease and arthritis (Huang et al., 2012; Perrier and Rutgeerts, 2012), and serve as an attractive future solution to reduce reproductive pathologies induced by genital chlamydial infection. Second, since TNF-α is induced early during the infection, this may serve as an early biomarker of disease development. A subset of individuals who contract genital chlamydial infections develop reproductive tract sequelae, but at present, no biomarkers have been identified to accurately predict impending disease in infected individuals. Third, this has implications to ongoing vaccine development efforts. The correlation between frequencies of IFN-γ and TNF-α producing CD4^+^ T cells and chlamydial clearance has recently been suggested as a justification to target the induction of *Chlamydia*-specific TNF-α, along with IFN-γ, for an efficacious anti-chlamydial vaccine (Yu et al., 2011). While a contribution of TNF-α to chlamydial clearance cannot be fully excluded, the finding from our study that kinetics of resolution of primary and secondary genital chlamydial infection was indistinguishable from WT, even in the total absence of TNF-α production, suggests that induction of a TNF-α response is not a necessity for anti-chlamydial protective immunity. Furthermore, the evidence of a role for TNF-α in chlamydial pathogenesis is compelling. Collectively, the contributions of TNF-α in genital chlamydial infections in context of both protective immunity and pathogenesis should be considered to achieve a safe and efficacious anti-chlamydial vaccine.

### Conflict of interest statement

The authors declare that the research was conducted in the absence of any commercial or financial relationships that could be construed as a potential conflict of interest.
